# Insights into the Hexose Liver Metabolism—Glucose versus Fructose

**DOI:** 10.3390/nu9091026

**Published:** 2017-09-16

**Authors:** Bettina Geidl-Flueck, Philipp A. Gerber

**Affiliations:** Division of Endocrinology, Diabetes, and Clinical Nutrition, University Hospital Zurich, 8091 Zurich, Switzerland; philipp.gerber@usz.ch

**Keywords:** NAFLD, type 2 diabetes, fructose, glucose, insulin, ChREBP, SREBP1c, gluconeogenesis, de novo lipogenesis, triglyceride

## Abstract

High-fructose intake in healthy men is associated with characteristics of metabolic syndrome. Extensive knowledge exists about the differences between hepatic fructose and glucose metabolism and fructose-specific mechanisms favoring the development of metabolic disturbances. Nevertheless, the causal relationship between fructose consumption and metabolic alterations is still debated. Multiple effects of fructose on hepatic metabolism are attributed to the fact that the liver represents the major sink of fructose. Fructose, as a lipogenic substrate and potent inducer of lipogenic enzyme expression, enhances fatty acid synthesis. Consequently, increased hepatic diacylglycerols (DAG) are thought to directly interfere with insulin signaling. However, independently of this effect, fructose may also counteract insulin-mediated effects on liver metabolism by a range of mechanisms. It may drive gluconeogenesis not only as a gluconeogenic substrate, but also as a potent inducer of carbohydrate responsive element binding protein (ChREBP), which induces the expression of lipogenic enzymes as well as gluconeogenic enzymes. It remains a challenge to determine the relative contributions of the impact of fructose on hepatic transcriptome, proteome and allosterome changes and consequently on the regulation of plasma glucose metabolism/homeostasis. Mathematical models exist modeling hepatic glucose metabolism. Future models should not only consider the hepatic adjustments of enzyme abundances and activities in response to changing plasma glucose and insulin/glucagon concentrations, but also to varying fructose concentrations for defining the role of fructose in the hepatic control of plasma glucose homeostasis.

## 1. Introduction

Eating habits and lifestyle are subject to change and are determined by socio-economic factors. In modern societies, the surplus offer of food leads to an excess caloric intake that may go along with reduced physical activity. The consequences are obesity with associated health risks such as nonalcoholic fatty liver disease (NAFLD) or type 2 diabetes. The continuously increased fructose consumption as sucrose and high fructose corn syrup over the past decades coincides with the raised prevalence of obesity, NAFLD, and type 2 diabetes [1,2,3]. These diseases in the end may lead to severe comorbidities as liver fibrosis/cirrhosis and consecutive failure of liver function (in the case of NAFLD), or severe cardiovascular disease as well as kidney, eye, and neurological disease in the case of diabetes. This coincidence led to a wide debate about the potential health risks of fructose consumption. It is beyond debate that the consumption of food, and especially beverages rich in fructose content, is often associated with an excessive energy intake. However, it is still an open question whether fructose exerts a negative effect on metabolism independently from effects on body weight in an energy balanced diet [4,5,6]. 

Studies about different forms of nutrition point out the importance of a food mix adjusted to our metabolic/biochemical disposition. During evolution, the human metabolism became adapted to gain energy from different sources for survival in an environment with a seasonally changing food offer. It is prepared to gain energy from a variety of simple and complex carbohydrates (consisting of a range of sugar types) as well as from fatty acids. The organism provides cellular pathways to obtain flexibility in the use of various energy carriers. However, may an unbalanced diet stretch cellular regulatory mechanisms to their limits and eventually impair health? What is the optimal mix of energy carriers for the human organism in terms of type of carbohydrates and fat for preserving health? There is no widely accepted paradigm in this respect.

The liver, as a central organ in carbohydrate metabolism, serves as an energy hub. It represents a major sink for carbohydrates. Hepatic glucose uptake contributes to the restoration of blood glucose homeostasis after a meal. Carbohydrates are stored as glycogen or converted into fatty acids which are then released into the plasma and deposited in adipose tissue. During fasting, glucose from the breakdown of glycogen, and gluconeogenesis is released by the liver maintaining blood glucose homeostasis. Impairment of such essential hepatic functions manifests as disturbed insulin sensitivity, increased hepatic glucose production and de novo lipogenesis. 

This article aims at highlighting the role of different types of sugars and their potential specific adverse effects on the liver. We refer to data from animal studies that examined diet induced gene expression and underlying molecular mechanisms, but we also included data from nutritional human studies investigating metabolic outcome based on tracer methods. We focus on the interconnections of fructose and glucose metabolism. 

## 2. Absorption and Distribution of Hexoses

Prior to going into the details of liver lipid metabolism and its adaptation to carbohydrate rich diets, some general differences between glucose and fructose and their potential roles as competitors need to be pointed out. 

It cannot be stressed enough that the cellular absorption and distribution of glucose and fructose differ markedly. Only two transporters, namely GLUT2 and GLUT5, of the 14 members of the glucose transporter (GLUT) family are permeable for fructose [7,8]. They are responsible for fructose uptake by facilitated diffusion. GLUT2 transporter is expressed in intestinal and kidney epithelial cells, on pancreatic beta cells, and hepatocytes [9]. In pancreatic beta cells, increased blood glucose levels induce insulin secretion. GLUT2 has also been attributed to a glucose sensing role on brain cells [10]. GLUT5 has an exclusive specificity for fructose [11,12]. Its main expression site is the apical membrane of intestinal epithelial cells providing a major route for absorption of dietary fructose [11,13,14].

GLUT5 expression is induced during the postnatal development. Its expression is dramatically stimulated by the introduction of dietary fructose and it seems to be modulated by paracrine and endocrine signals leading to a diurnal variation of expression in adults [15,16,17,18]. Fructose enters the epithelial intestinal cells via the GLUT5 transporter by the mechanism of facilitated diffusion, whereas glucose enters the enterocytes via sodium-glucose-cotransporter 1, an active transporter [13,19]. Further, GLUT2 may contribute to intestinal fructose and glucose absorption, although this is to a minor extent [20,21,22,23]. In intestinal cells, a proportion of fructose is supposed to be converted into glucose as enzymes of the gluconeogenetic pathways are induced by intestinal fructose [24]. Fructose and glucose are released into the portal vein via GLUT2 that is located in the basolateral membrane of enterocytes. Consecutively, a large proportion of fructose is taken up postprandially by the liver representing its major sink [25]. However, skeletal muscle, adipose tissue, and the kidney also contribute to fructose clearance from the blood [26,27,28,29]. Glucose is distributed into different tissues according to the wide expression of different glucose transporters expressed by most mammalian cells [7]. Most important for postprandial whole body glucose disposal are muscle cells that take up glucose via GLUT 4, which is upregulated by insulin [30].

Hepatic uptake of both glucose and fructose is mediated by the GLUT2 transporter, which is constitutively expressed on hepatocytes and facilitates diffusion [9]. The affinity of GLUT2 for glucose is higher than for fructose (Km for glucose is ~17 mM and for fructose ~76 mM) [31].

After hepatic uptake, fructose and glucose are phosphorylated by different kinases. In the liver, fructose is phosphorylated by the high-activity keto-hexokinase isoform C (KHK-C), which is more effective than the ubiquitous low-activity KHK-A, and subsequently rapidly converted into trioses [32]. The rapid processing of entering fructose is maintaining the fructose concentration gradient between plasma and hepatocytes that leads to a continuous uptake of fructose into the hepatocyte [33,34]. The importance of KHK for the uptake of fructose is not only reflected by the fact that selective knock-out of KHK-A isoform may enhance fructose influx to the liver, but also that a switch from the KHK-A to the KHK-C isoform enhances fructose metabolism, whereas a switch from KHK-C to KHK-A reduces fructose metabolism [35,36]. KHK is neither feedback-inhibited nor allosterically regulated [37]. In contrast, glucose is phosphorylated by glucokinase (GK), whose transcription is induced by insulin and which is acutely regulated by GK regulatory protein (GKRP) [38,39,40,41]. GKRP’s inhibitory effect on GK is increased by fructose-6-phosphate (F6P) and suppressed by fructose-1-phosphate (F1P) [42,43,44]. Changes in GK activity due to genetic mutations are associated with a distinct form of late onset diabetes characterized by impaired hepatic glucose disposal and increased glucose production [45]. There is no direct allosteric feedback inhibition by glucose-6-phosphate (G6P) on GK [46]. However, the allosteric activation of glycogen synthase (GS) by G6P may increase glucose influx [47]. The phosphorylation rate by KHK is 10 times higher than by GK [48].

In summary, hepatic influx rates of fructose and glucose are determined by their plasma concentrations and GLUT2 affinities, but also by the KHK activity for fructose and the GK and GS activity for glucose. 

## 3. How do Fructose and Glucose Modulate the Hepatic Uptake and Metabolism of Each Other?

The hepatic response to rising intracellular hexose concentrations is orchestrated by hormones, key regulatory transcription factors, and metabolic intermediates that adjust both enzyme abundances and activities. The relative importance of the regulation of enzyme abundances and activities, respectively, in the control of the metabolic hexose flux and finally the restoration and maintenance of glucose homeostasis is debated. However, the results from mathematical modeling suggest that the regulation of enzyme abundances and of enzyme activities is equally important for the regulation of hepatic hexose metabolism [49].

**Figure 1:**
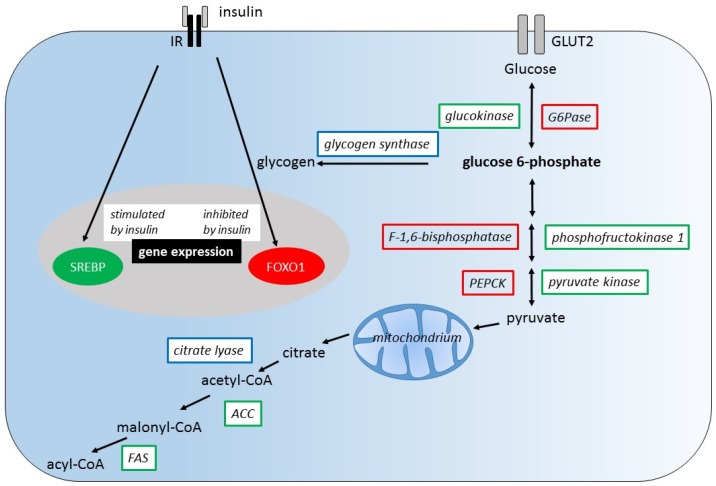
Regulation of liver metabolism by insulin. Insulin stimulates the expression of genes encoding glycolytic and lipogenic enzymes (green), but inhibits the expression of those encoding gluconeogenic enzymes (red). The induction of gene expression by insulin is mediated by the transcription factor sterol regulatory element binding protein (SREBP), the repression of expression is mediated by forkhead box O1 (FOXO1). Insulin also regulates the activity of glycogen synthase and citrate lyase (blue). Insulin activates glycogen synthase promoting its dephosphorylation through inhibition of protein kinase A (PKA) and glycogen-synthase-kinase-3 (GSK-3). IR, insulin receptor; GLUT2, glucose transporter 2; PEPCK, phosphoenolpyruvate carboxykinase; ACC, acetyl-CoA carboxylase; FAS, fatty acid synthase (Adapted from [51]).

Certainly, a comparison of hepatic metabolism of glucose and fructose requires the consideration of the diverse actions of insulin in order to identify possible sites of interaction of glucose and fructose metabolism. We cover this issue by starting from the widely accepted actions of insulin on liver metabolism namely blocking glycogenolysis and gluconeogenesis, stimulating glycogen synthesis and thus regulating fasting glucose levels (illustrated in Figure 1) and aim to highlight fructose specific effects that may enhance or antagonize the effects of insulin [50].

On one hand, insulin directly stimulates the expression of glycolytic enzymes such as GK, phosphofructokinase 1 (PFK1), and pyruvate kinase (PK) and inhibits the expression of gluconeogenic enzymes such as phosphoenolpyruvate carboxykinase (PEPCK), F-1,6-bisphosphatase (F1,6BPase), and glucose-6-phosphatase (G6Pase). The regulatory mechanisms of gene expression by insulin involve sterol regulatory element binding protein 1c (SREBP1c) and forkhead box O1 (FOXO1). On the other hand, the immediate action of insulin is the regulation of enzyme activities namely of GS and citrate lyase by changing their phosphorylation state. 

**Figure 2:**
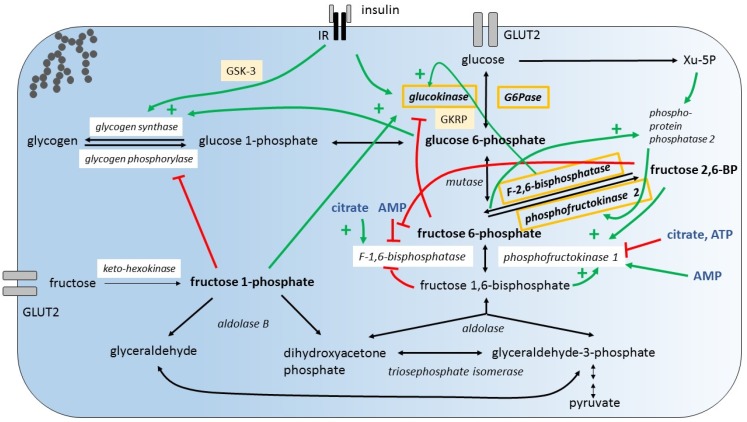
Hepatic hexose uptake and regulatory mechanisms that control hexose breakdown. Glucose and fructose is taken up by glucose transporter 2 (GLUT2). Fructose metabolites regulate key enzymes of glucose metabolism. (A) Fructose stimulates glucose uptake by distinct mechanisms:(1) Fructose 1-phosphate (F1P) antagonizes the inhibition of glucokinase (GK) by glucokinase regulatory protein (GKRP) promoting glucose 6-phosphate (G6P) production; (2) newly synthesized G6P stimulates glycogen synthase; (3) F1P inhibits glycogen phosphorylase. (B) Fructose 6-phosphate (F6P) and xylulose 5-phosphate (Xu-5P) activate phosphoprotein-phosphatase 2 which activates phosphofructokinase 2 promoting generation of fructose 2,6-bisphosphate (F2,6BP). F2,6BP activates phosphofructokinase 1 and inhibits F-1,6-bisphosphatase. (C) Insulin signaling increases glucokinase expression and promotes glycogen synthesis enhancing glycogen-synthase-kinase-3 (GSK-3) phosphorlyation. (D) From metabolic control analysis it is concluded that in the fed, hyperglycemic state, glucose exchange flux is controlled by GK, fructose-2,6-bisphosphatase, glucose 6-phosphatase (G6Pase) and phosphofructokinase-2 (orange box), while in the fasted, hypoglycemic state pyruvate carboxylase, and lactate transporter control the glucose exchange flux [49]. IR, insulin receptor; AMP, adenosine mono-phosphate; ATP, adenosine tri-phosphate. For completeness, the illustration contains also some additional regulatory mechanisms not discussed in the article.

Hepatic glucose metabolism is not only subject to insulin-dependent regulation, as a variety of glucose intermediates as well as adenosine mono-phosphate (AMP), adenosine tri-phosphate (ATP), and citrate are known to be potent direct or indirect modulators of glycolytic, gluconeogenic, and glycogenic enzyme activities. Fructose specific intermediates also play an important role in the regulatory system of glycolysis/gluconeogenesis and glycogenesis [33]. To be mentioned are the allosterically regulated glycolytic enzymes GK, PFK1, and the gluconeogenic enzymes F1,6BPase and glycogenic enzymes GS (Figure 2).

### 3.1. Modulation of Glucose Uptake and Glycogenesis by Fructose

Small amounts of dietary fructose have been shown to dramatically increase the hepatic glucose uptake through GK activation [52,53]. It is proposed that fructose 1-phosphate (F1P) antagonizes the inhibition of GK by GKRP [44,54]. The allosteric activation of GS by increased G6P concentrations and the simultaneous inhibition of glycogen phosphorylase by F1P may generate a glucose influx into the hepatocyte [55,56] (Figure 2 shows hepatic glucose and fructose metabolism and illustrates major regulatory mechanisms). A positive impact of small amounts of fructose on glycemic control, and in particular on postprandial glucose levels, but also on long-term glycemic control (HbA1c) has been reported in different human studies and meta-analyses with healthy individuals or patients with diabetes [57,58,59,60,61,62]. In respect to glucose uptake and the formation of glycogen, small amounts of fructose are contributing to the action of insulin. 

However, the excess consumption of fructose (corresponding to the 90th percentile of fructose intake of the US adults and above) has been shown to impair glucose uptake, reducing GK activity and liver glycogen content [63]. While insulin signaling and GK mRNA levels remained unaffected by fructose ingestion, GK and GKRP protein content was decreased. The underlying mechanisms are not known. However, it is suggested that excess fructose consumption induces a significant posttranscriptional decrease in GK protein levels. In respect to glucose uptake and formation of glycogen, large amounts of fructose are antagonizing the action of insulin.

### 3.2. Conversion of Fructose into Glucose/Glycogen

**Figure 3:**
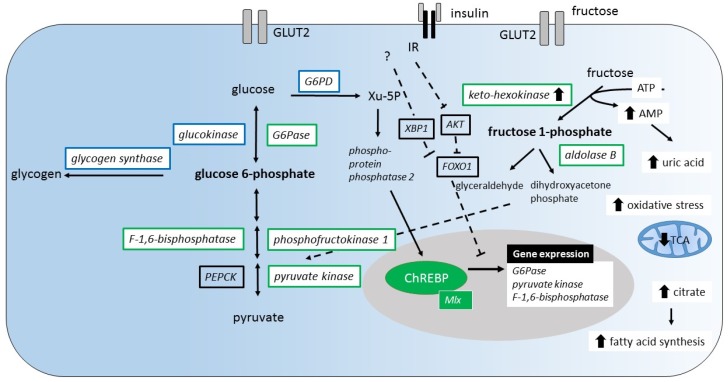
Changes of hepatic gene expression and metabolic pathways induced by fructose. (A) Fructose stimulates the expression of the fructose metabolizing enzymes (keto-hexokinase and aldolase B), glycolytic enzymes (phosphofructokinase 1, pyruvate kinase), but also the expression of gluconeogenic enzymes (glucose 6-phosphatase (G6Pase), F-1,6-bisphosphatase, pyruvate kinase (green)). These effects are thought to be mediated by transcription factor carbohydrate responsive element binding protein (ChREBP). Fructose is suggested to increase xylulose-5 phosphate (Xu-5P) levels, which leads to nuclear translocation of ChREBP. It is suggested that fructose induced ChREBP activation dominates over the insulin and/or X-Box Binding Protein 1 (XBP-1) suppressive effects mediated by forkhead box O1 (FOXO1) inhibition. (B) Increasing levels of uric acid from adenosine mono-phosphate (AMP) catabolism increase mitochondrial oxidative stress. The resulting inhibition of tricarboxylic acid cycle (TCA) increases the mitochondrial citrate release stimulating fatty acid synthesis. IR, insulin receptor; G6PD, glucose 6-phosphate dehydrogenase; PEPCK, phosphoenolpyruvate carboxykinase; ATP, adenosine tri-phosphate.

It has been demonstrated that the consumption of dietary fructose compared to glucose induces increased expression of the fructose metabolizing enzymes KHK, aldolase B and the glycolytic enzymes PFK1 and PK [64] (Figure 3). Among the gluconeogenic enzymes, F1,6BPase and G6Pase are specifically induced by dietary fructose. Yet, the underlying mechanism of fructose-induced gene expression is unknown. However, a ChREBP driven mechanism that works independently from FOXO1 signaling is suggested to induce G6Pase and F1,6BPase expression [64,65]. Based on these findings, one may conclude that insulin and fructose exert the same enhancing effect on the expression of a subset of glycolytic enzymes namely PFK1 and PK. However, with respect to gluconeogenic enzymes F1,6BPase and G6Pase insulin and fructose show opposing effects. Additional data about the effects of co-ingestion of glucose with fructose on the expression of gluconeogenic and glycolytic enzymes are not available.

Simultaneous enhancement of glycolytic enzyme expression by insulin and fructose, while opposing effects on the expression of gluconeogenic enzymes appears paradoxical and raises the question about the metabolic outcome when fructose and glucose are co-ingested. Does dietary fructose promote futile cycling at the site of conversion of F6P into F-1,6-bisphosphate (F1,6BP) and vice versa (Figure 2) [66]? What are the effects of key intermediates such as fructose-2,6-bisphosphate (F2,6BP), AMP and ATP? One important regulatory step during glycolysis is the conversion of F6P into F1,6BP during glycolysis by PFK1 and the conversion of F1,6BP into F6P by F1,6BPase during gluconeogenesis, respectively. While PFK1 is inhibited by high levels of ATP and induced by increased levels of F2,6BP and F1,6BP, F1,6BPase is induced by high citrate levels and inhibited by increased F2,6BP, F1,6BP, and AMP levels. Thus, increased levels of F2,6BP generated by phosphorylation of F6P by PFK2, F1,6BP, and AMP promote glycolysis. F6P activates phosphoproteinphosphatase 2, which induces the kinase activity of the bifunctional enzyme phosphofructokinase-2/fructose-2,6-bisphosphatase (PFKFB) and thereby increases F2,6BP levels [67]. The relative impact of regulation of the expression of key enzymes and their activity on the metabolic outcome is unknown. However, the metabolic outcome of fructose consumption with respect to gluconeogenesis has been studied using tracer-based methods. There is actually significant gluconeogenesis from fructose also when co-ingested with glucose [68,69]. The impact of fructose consumption on blood glucose concentration and glycemic control has been the subject of extensive meta-analyses [70,71].

Newly generated glucose from fructose is released into the plasma, but is there also deposition in the glycogen storage? Glycogen deposition depends on the activity of GS that catalyzes the rate limiting step in glycogen synthesis and the activity of glycogen phosphorylase. It is known that insulin and G6P activate the GS and F1P inhibits glycogen phosphorylase. A further tracer study showed that fructose contributes little to glycogen synthesis when ingested alone (*n* = 6, healthy volunteers). However, if co-ingested with glucose, fructose contributes significantly to glycogen synthesis suggesting a critical role of insulin [69]. Insulin stimulates the expression of GK and activates GS that catalyzes the rate limiting step in glycogen synthesis [72,73,74]. 

The described mechanisms explain how fructose and glucose may act as molecules that modulate the metabolic fate of each other. Both energy carriers compete for glycogen storage capacity and access to the tricarboxylic acid cycle (TCA) and fatty acid synthesis pathways. 

Fructose was primarily described as “lipogenic substrate” in the context of hepatic metabolism, however, studies demonstrate that gluconeogenesis and glycogen deposition also play an important role in fructose metabolism. The metabolic effects of fructose consumption are dose-dependent. Small amounts of fructose may substantially enhance the uptake of glucose into the liver and glycogen deposition, while large amounts of fructose may reduce the hepatic glucose uptake. The presence of insulin determines the fate of fructose. The fact that co-ingestion with glucose directs fructose to the glycogen storage probably through the action of insulin would suggest that glucose takes temporarily the load off the other fructose disposal pathways.

## 4. Lipogenesis

De novo lipogenesis (DNL) is the conversion of carbohydrates or proteins into fatty acids, enabling the organism to store energy in an unlimited way as fat. DNL takes place in the cytosol of hepatocytes and adipocytes if there is an excess of the substrate acetyl co-enzyme A. Critical enzymes of the synthesis pathway of fatty acids are the Acetyl CoA Carboxylase (ACC), the fatty acid synthase (FAS), elongation of long-chain fatty acids family member 6 (ELOVL6), as well as stearoyl-CoA desaturase 1 (SCD1) [75,76,77,78]. Increased availability of acetyl co-enzyme A substrate and elevated enzyme activities and quantities have been shown to directly enhance the output of newly synthesized fatty acids, such as palmitate and stearate. As illustrated above, studies point out that fructose probably increases the levels of lipogenic substrates more as compared to glucose ingested alone. Fructose as well as glucose induce transcription of lipogenic enzymes. The question of whether lipogenic enzymes are induced directly by carbohydrates or indirectly by the stimulation of insulin secretion raises the issue of sugar specific impacts on liver metabolism. Are there any hexose specific processes regulating lipogenesis?

### 4.1. Transcriptional Regulation of the Expression of Lipogenic Enzymes by Carbohydrates

There are several master transcription factors regulating the expression of partly the same set of lipogenic enzymes. ChREBP induces the transcription of FAS, SCD1, ELOVL6, ACC, and PK [79,80]. SREBP1c upregulates the expression of FAS, ACC, stearoyl-CoA desaturase 1 SCD1, ELOVL6 [81,82]. X-Box Binding Protein 1 (XBP-1), known as mediator of the endoplasmic reticulum (ER) stress response, has been attributed an additional function as a transcription factor of the lipogenic genes FAS, SCD1, ACC 1 and ACC 2 [83].

#### 4.1.1. ChREBP

**Figure 4:**
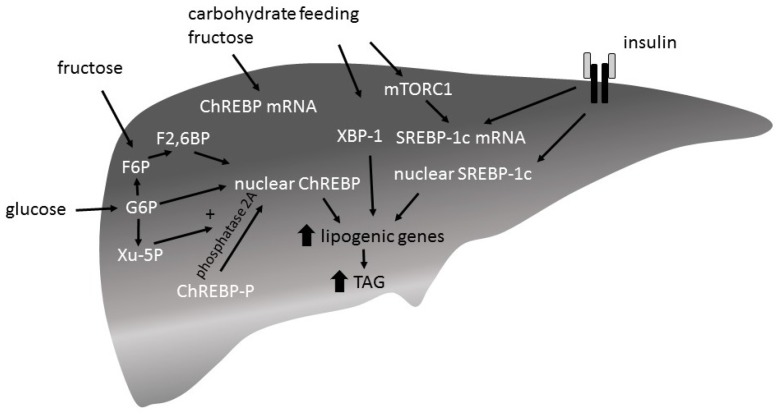
Carbohydrate induced expression of lipogenic genes. (1) Activation of carbohydrate responsive element binding protein (ChREBP): xylulose 5-phosphate (Xu-5P), glucose 6-phosphate (G6P), and fructose-2,6-bisphosphate (F2,6BP) promote nuclear entry of ChREBP. (2) Sterol regulatory element binding protein-1c (SREBP-1c) transcription and dependent expression of lipogenic enzymes is mediated by mechanistic target of rapamycin C1 (mTORC1) signaling. mTORC1 is induced by insulin signaling, but also directly by carbohydrate feeding. Translocation of SREBP1c depends on insulin signaling. (3) X-Box Binding Protein 1 (XBP-1) is involved in the control of lipogenic gene expression. The underlying mechansims are unknown.

Hepatic ChREBP expression is induced by a high-carbohydrate diet [79,84]. Feeding studies comparing the potency of fructose versus glucose to induce ChREBP expression have shown different outcomes: two studies report that fructose more potently induced ChREPB expression [64,65], whereas another study reported no differences [85]. Although it is suggested that insulin too is implicated in the regulation of ChREBP expression [86], liver insulin receptor knock-out mice show an increased ChREBP expression after ad libitum chow feeding [85]. There are two ChREBP isoforms identified: ChREBPα and ChREBPβ. The specific roles of the isoforms are not yet clear. It is thought that glucose first induces the expression of ChREBPα by inducing the transcription of ChREBPβ, which in turn is supposed to be the more potent isoform [86,87]. While only little is known about the transcriptional regulation of ChREBP, post-translational regulation of ChREBP has extensively been investigated (Figure 4). Intermediates of the glycolytic pathway and the pentose pathway play an important role in the regulation of ChREBP activity [88,89,90]. Entrance into the nucleus and binding to the DNA binding site CHoRE are regulated by G6P, xylulose 5-phosphate (Xu-5P), and F2,6P via the post-translational modification of ChREBP enhancing DNA binding and transcriptional activity. 

Overall, any hexose pathway that ends in the gluconeogenetic pathway at the level of trioses (dihydroxyacetone phosphate and glyceraldehyde) potentially enhances ChREBP dependent expression of lipogenic enzymes. Fructose may directly activate ChREBP through fructose derived intermediates or indirectly through activation of GK enhancing the glycolytic flux. The importance of ChREBP for the regulation of hexose metabolism is reflected in ChREBP deficient mice which show glycogen accumulation and decreased triglyceride (TAG) synthesis in the liver [91]. 

#### 4.1.2. SREBP1c

There are three SREBP isoforms, SREBP-1a, -1c and 2. SREBP1c is known to induce lipogenic gene expression and its expression itself is induced by carbohydrate feeding. Ad libitum chow-feeding of mice (24 h, fed state) increased SREBP1c expression in wildtype mice and to a lesser extent also in liver insulin receptor knock-out mice (LIRKO) [85]. SREBP1c expression is known to be regulated by a mechanistic target of rapamycin C1 (mTORC1), which is activated by carbohydrate feeding as well as by insulin signaling. It is thought that insulin is necessary for a maximal induction of SREBP1c expression by carbohydrate feeding.

The potential of fructose to induce SREBP1c expression has also been examined in mice. The impact of a 1-week high-fructose diet (60% fructose) was compared to a standard chow diet on SREBP1c expression. Fructose diet increased SREBP1c mRNA levels and consequently the mRNA levels of lipogenic enzymes more potently than standard chow diet in wildtype, as well as in LIRKO mice. Levels of nuclear SREBP1c protein in LIRKO mice are lower than in wildtype mice after both fructose and standard chow diet indicating the important role of insulin signaling.

Insulin binding to the insulin receptor activates the phosphoinositid-3-kinase/protein kinase B (PI3K/PKB) pathway and results in the proteolytic activation of SREBP1c through the interaction of SCAP (SREBP cleavage-activating protein) and INSIG1 (insulin-induced gene-1), and its release from the Golgi apparatus [92]. Mice with conditional SCAP deficiency in the liver (L-SCAP-mice) and therefore no functional SREBP isoforms show a reduced hepatic FA synthesis [93]. Livers of these mice show reduced mRNA levels of lipogenic enzymes and do not respond with an increase in mRNA levels upon insulin stimulation. Their very low density lipoprotein (VLDL) levels in the plasma are reduced. In L-SCAP-mice, there is a compensatory increase in fatty acid synthesis in adipose tissue [94]. 

#### 4.1.3. XBP-1

XBP-1 is an active transcription factor which increases the ER folding capacity in response to ER stress (unfolded protein response (UPR)) [83]. Apart from this central role, XBP-1 has been identified as regulator of hepatic DNL. Specific deletion of XBP-1 in the adult liver reduces plasma TAG and is thought to reduce lipids synthesis in the liver [95]. In wildtype mice but not XBP-1 knock-out mice, carbohydrate feeding induces hepatic expression of FAS, SCD1, ACC 1, and ACC 2. A high-fructose diet compared to a standard chow diet induces the expression XBP-1 more potently. The signal that activates XBP-1 upon carbohydrate feeding and how/whether it relates to ER stress is unknown [83]. It has been shown that XBP-1 inhibits FOXO1 signaling suppressing the expression of gluconeogenic enzymes [96].

Although mechanisms are described that involve insulin in the regulation of the expression of both ChREBP and SREBP1c, feeding studies with insulin receptor knock-out mice show that insulin is required neither for the induction of ChREBP nor for SREBP1c. However, in the case of SREBP1c the presence of insulin is required for a full induction of SREBP1c expression upon carbohydrate consumption. ChREBP, SREBP1c and XBP-1 are more potently induced by a high-fructose compared to high-glucose diet [64,65,83,85]. Consequently, lipogenic gene expression is also significantly more increased by fructose compared with a glucose diet. Overall, there is evidence that fructose is more potent than glucose in respect to the induction of lipogenic gene expression. 

### 4.2. Stimulation of DNL by Fructose via Purine Degradation 

Rapid phosphorylation of fructose by KHK decreases ATP levels, paralleled by an increase in AMP levels [97,98]. It has been shown that AMP enters into the purine degradation pathway through the activation of AMP deaminase resulting in uric acid production and the generation of mitochondrial oxidants. There is evidence that fructose via a purine-degrading pathway stimulates triglyceride synthesis [97]. The inhibition of aconitase within the TCA cycle by mitochondrial oxidative stress leads to the accumulation of citrate, the stimulation of ATP citrate lyase, and fatty-acid synthase leading to DNL.

### 4.3. Stimulation of Fatty Acid Synthesis by Carbohydrates—Results from Isotopic Tracer Studies

It is an open question, to which extent de novo lipogenesis (DNL) from carbohydrates is contributing to TAG synthesis/secretion or eventually to the TAG accumulation in hepatocytes. In the past, studies have addressed this issue by using isotope-based methods for the measurement of de novo lipogenesis. After the consumption of different kinds and amounts of sugars, rates of secretion of newly synthesized palmitate, or the fractional de novo lipogenesis (the proportion of newly synthesized palmitate of the totally secreted palmitate) have been calculated in different experimental settings. Here, two studies should be mentioned that applied the mass isotopomer distribution analysis (MIDA) approach for which 13C-acetate is infused and 13C-palmitate isotopomers from isolated VLDLs are measured. In one study, the short-term effects of a hypercaloric carbohydrate (hydrolyzed corn starch) feeding on fatty acid synthesis were examined in healthy men (*n* = 5). A hypercaloric carbohydrate diet (around 2.5 times energy expenditure) during four days generated moderate hyperinsulinemia and increased VLDL-TAG concentrations. It caused an increase in the secretion rate of VLDLs, newly synthesized palmitate and preformed fatty acids, and a decreased VLDL-TAG catabolism [99]. The other study compared the short-term effects of an energy-balanced high-fructose diet (25% energy intake) with the effects of a complex carbohydrate diet with low-fructose (5% energy intake) on fractional DNL in healthy men (*n* = 8). After six days of this diet, there was no significant difference in respect to the fasting fractional DNL [100]. In contrast, postprandial fractional DNL after 9 days of diet indicated as integrated DNL (area under the curve) was increased during the high-fructose diet when compared with the complex carbohydrate diet. A further study demonstrated that moderate aerobic exercise neutralizes the effects of an energy-balanced high-fructose diet on postprandial TAG concentrations and DNL [101]. It was speculated that DNL was decreased due to a reduced availability of fructose substrate for lipogenesis during exercise. 

The focus of further studies should be on the long-term effects of free fructose versus free glucose on DNL in energy-balanced diets. They should aim for the determination of the rate of secretion of newly synthesized and preformed FA induced by high-glucose vs. -fructose diets. Further information about the impact of sugar type on liver metabolism is needed. 

## 5. Fatty Acid Oxidation 

During the fasted state, mitochondrial fatty acid oxidation produces energy in the liver. Beta oxidation provides energy for the hepatocyte and also ketone bodies that are exported to extrahepatic tissues. Beta oxidation is regulated at several stages.

Peroxisome proliferator-activated receptor alpha (PPAR α) is a master regulator of hepatic lipid metabolism and its expression is induced in the fasted state [102]. It is induced by glucagon and it is activated by a subtype of longchain fatty acids (LCFA) and phospatidylcholines [103,104].

PPAR α controls the fatty acid import into and processing within the mitochondria by carnitine palmitoyltransferase 1 (CPT-1) and important enzymes of the beta oxidation pathway, such as acyl-CoA dehydrogenases (Acad) [105,106]. Fructose has been shown to decrease beta oxidation by downregulation of PPARα [107]. Dietary unsaturated fatty acids showed a favorable effect on hepatic gene expression mediated by PPARα and mimic the effect of synthetic PPARα agonists [108].

The rate limiting step of beta oxidation is the translocation of LCFA into the mitochondrium mediated by CPT-1. Malonyl-CoA, produced by carboxylation of acetyl CoA by ACC2, is inhibiting CPT-1. Malonyl-CoA is the primary substrate for lipogenesis and by inhibiting CPT-1 it avoids a futile cycle [109,110,111,112]. Ongoing fatty acid synthesis from carbohydrates interferes with the beta oxidation via malonyl-CoA. 

The effect of low- and high-carbohydrate diets on substrate oxidation has been investigated in healthy men (*n* = 5) by using stable-isotope methodology and indirect calorimetry. Two weeks of a high-carbohydrate diet (75% carbohydrates, 10% fat, 15% protein) significantly reduced the total and plasma fatty acid oxidation when compared to a 2-week low-carbohydrate diet (30% carbohydrates, 55% fat, 15% protein) [113]. It was shown that there is a carbohydrate-induced inhibition LCFA entry into the mitochondria using tracer methodology [114]. Oxidation of 13C-LCFA and 14C-medium-chain fatty acid (MCFA) was measured during hyperglycemic and hyperinsulinemic clamps. Entrance of LCFA but not of MCFA into the mitochondria is known to depend on CPT 1. Only the oxidation of LCFA but not of MCFA was significantly reduced. This suggests that carbohydrates inhibit fatty oxidation by inhibition of CPT1/entrance of LCFA into the mitochondria. To our knowledge, there is no study directly comparing the effects of high-fructose and -glucose energy balanced diets on fatty acid oxidation.

## 6. TAG Synthesis and VLDL Secretion

The primary determinant for TAG synthesis and secretion is the availability of free fatty acids in the liver [115,116]. Fatty acids (FA) in the liver are of different origin [117,118]. They can be non esterified fatty acids (NEFA) released into the plasma by adipocytes, fatty acids newly synthesized by the liver, or dietary fatty acids. The proportion of FA from the different sources depends critically on the metabolic state. During the fed state, the proportion of NEFA from adipose tissue declines as lipolysis is turned off by insulin. In contrast, DNL is increased as lipogenic substrates are available from carbohydrate catabolism and by the stimulatory effect of insulin on fatty acid synthesis. Newly synthesized malonyl-CoA suppresses fat oxidation. Postprandially, the dietary fatty acids in the liver increase due to the spillover of lipoprotein lipase (LPL)-generated fatty acids and TAG delivery by chylomicron remnants [119]. In the fasted state, the proportions are reversed. FA from lipolysis increases, DNL declines and the supply of dietary fatty acids ceases. 

The rate of the hepatic uptake of NEFAs is given by the plasma NEFA concentration and the amount and activity of transporter protein [120]. NEFAs from the plasma enter the hepatocyte mainly via fatty acid transport protein (FATP) and are activated to fatty acid CoA. FA are activated to acyl-CoA in the hepatocyte in a largely unknown process. Three acyl-CoA from entered fatty acids/preformed fatty acids or newly synthesized fatty acids are sequentially esterified involving different transferases (glycerol phosphate acyltransferase (GPAT), acylglycerolphosphate acyltransferase (AGPAT), diacylglycerolphosphate acyltransferase (DGAT)) to a glycerol molecule to build TAG. TAGs are packaged with apoB 100 to VLDL in the ER by the microsomal TAG transfer protein (MTP). Insulin acts as a regulator of VLDL assembly and secretion. Insulin induces apolipoprotein B 100 (ApoB 100) degradation limiting the postprandial secretion of VLDL [121].

The effects of fructose consumption on TAG concentrations has not been comprehensively clarified. On the one hand, there is evidence that carbohydrate and high-fructose intake significantly increase the blood TAG concentrations probably due to stimulation of DNL and VLDL secretion in weight-maintaining diets [100,122,123,124,125]. In addition, a high-fructose intake may impair postprandial TAG clearance possibly due to decreased insulin levels and consequently decreased the activity of lipoproteinlipase [126]. On the other hand, data from meta-analyses show that while isocaloric exchange for other carbohydrate does not increase postprandial triglycerides and has no negative effects on established lipid parameters [127], fructose providing excess energy does increase postprandial triglycerides. Longer and larger trials on this topic are needed—as concluded by a systematic review and meta-analysis of controlled feeding trials [128]. Current literature delivers quite heterogeneous results regarding the effect of isocaloric fructose exchange for carbohydrate on blood lipids [129]. 

**Figure 5:**
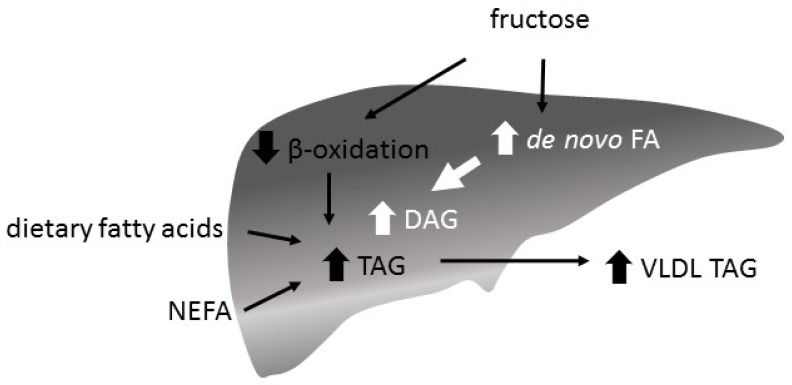
Hepatic triglyceride (TAG) synthesis and very low density lipoprotein (VLDL) secretion. The abundance of fatty acids (FA) in the liver is given by the plasma concentration of dietary FA and non esterified fatty acids (NEFA) and the amounts of newly synthesized FA and beta oxidation rate. It is the main determinant of the hepatic TAG/VLDL secretion rate. FA are sequentially esterified with glycerol by different transferases and secreted as VLDL. Fructose decreases beta oxidation and increases hepatic FA/TAG content and VLDL secretion.

A fructose-induced increase of de novo fatty acid synthesis and a decrease of beta oxidation is supposed to increase hepatic FA content and to enhance TAG synthesis and VLDL secretion (Figure 5). While a hypercaloric high-fructose diet is associated with an increase in intrahepatocellular lipid content (IHLC), isocaloric exchange of fructose for other carbohydrates does not increase IHLC in healthy participants [130]. However, there is also a study reporting an increased IHLC with a high-fructose energy balanced diet [100] 

## 7. Association of Chronic Fructose Consumption with Reduced Insulin Sensitivity and Continuous Hepatic Production of Glucose

Potential implications of high-fructose consumption on insulin sensitivity and blood lipids have been examined extensively. Fructose has generally been recognized as sugar that decreases insulin sensitivity and promotes dyslipidemia [131,132].

Focusing on the effects of fructose consumption in healthy men, a study by Aeberli et al. examined the implications of fructose ingestion in amounts close to usual daily consumption when soft drinks replace unsweetened beverages (*n* = 9). The study assessed the effects of moderate amounts of fructose and sucrose as compared with glucose on insulin sensitivity and lipid metabolism. Moderate amounts of fructose consumption impair insulin sensitivity in healthy young men, without affecting plasma glucose and TAG levels [133]. Of interest, it was in particular the suppression of hepatic glucose production that could be detected by the use of euglycemic-hyperinsulinemic clamp technique.

Further evidence that fructose consumption reduces hepatic insulin sensitivity and induces hyperglycemia comes from studies where hepatic fructose uptake was prevented. An animal study presenting a KHK knock-out mouse (KHK-A/C deletion) confirmed a pivotal role of KHK in the regulation of hepatic fructose uptake. The deletion of KHK abolished hepatic fructose uptake and led to a dramatic hyperfructosemia, but prevented fructose-induced hyperglycemia [134]. In particular, the impact of chronic fructose consumption on plasma glucose concentrations in KHK knock-out mice vs. wildtype mice was compared. After 12-weeks of ad libitum fructose feeding, there was fructose-induced hyperglycemia in wildtype mice with associated increased HbA_1c_ levels, but not in KHK-/- mice. 

**Figure 6:**
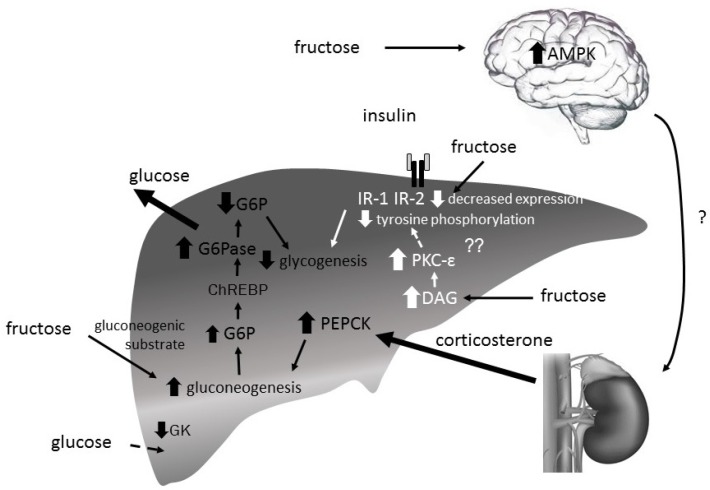
Proposed hypotheses explaining hepatic insulin resistance induced by fructose. (1) Increased diacylglycerols (DAG) levels activate (protein kinase C-ε) PKC-ε leading via a cascade of serine-threonine kinases finally to a decreased tyrosine phosphorylation of insulin receptor 1 and 2 (IR-1,-2) which interferes with insulin signaling. Consequently, glycogen synthase activity decreases. (2) Fructose-induced hypothalamic activation of 5′-AMP-activated protein kinase (AMPK) increases via an unknown effector corticosterone release by the adrenal gland. The resulting enhanced phosphoenolpyruvate carboxykinase (PEPCK) expression increases gluconeogenesis. (3) Fructose-induced glucose-6-phosphatase (G6Pase) activity has been identified as main determinant of hepatic glucose production dominating over the suppressive effects of insulin. (4) Excessive fructose consumption may induce a significant posttranscriptional decrease in glucokinase (GK) protein levels. (5) Fructose decreases IR-2 expression.

The precise mechanisms underlying fructose induced hepatic insulin insensitivity are still unknown. Lines of research currently focus on mechanisms driving gluconeogenesis by increasing expression of gluconeogenic enzymes namely PEPCK, F1,6BPase and G6Pase. Yet, there are also studies examining the impact of high-fructose diets on hepatic glucose uptake as well as glycogen formation [63] (Figure 6).

In rats, a long-term high-fructose, but not -glucose (eucaloric) diet caused hepatic deposition of TAG and impaired insulin signaling and glucose tolerance, but did not change the expression of PEPCK (fasted state) [135]. Also in mice, long-term high fructose as well as glucose feeding did not alter PEPCK expression (fed and fasted state) [64]. Another study investigated the acute effect of fructose on PEPCK expression. It is suggested that fructose consumption increases the hepatic PEPCK gene expression via the activation of hypothalamic 5′-AMP-activated protein kinase (AMPK) [136]. Fructose, at least in a rodent model, may increase corticosterone levels through a mechanism that is dependent on hypothalamic AMPK activation resulting in an increased hepatic PEPCK expression, although these conclusions remain speculative for the time-being. The role of PEPCK for fructose induced insulin sensitivity has not yet been conclusively defined.

Further studies suggest that fructose feeding increases the expression of the gluconeogenic enzymes F1,6BPase and also G6Pase driving hepatic gluconeogenesis and glucose release [64,65]. It is suggested that a carbohydrate-mediated ChREBP-driven FOXO independent mechanism induces F1,6BPase and G6Pase expression. In addition, it is hypothesized that an increased G6Pase activity may decrease hepatic G6P levels, which could stimulate glycogen breakdown. The resulting increase of hepatic glucose release could promote hyperinsulinemia and insulin insensitivity.

Furthermore, as mentioned earlier, excessive fructose consumption may lead to decreased GK levels, hepatic glucose uptake, and further decreased GS activity resulting in decreased glycogen deposition [63]. 

Overall, even though long-term fructose consumption (supplementation of eucaloric 10% *w*/*v* fructose or glucose solutions for 2 months, ad libitum fed rat) increased hepatic TAG content and decreased hepatic insulin receptor 2 (IR-2) expression and Akt activation, the impact on hepatic metabolism has to be further clarified [135]. Namely, whether it leads to decreased glycogen synthase activity due to decreased GSK-3 activity as reported for high fat diet induced-fatty liver remains elusive [137]. The impact of increased hepatic TAG fat has generally been associated with decreased insulin sensitivity. There are opposing data whether fructose consumption leads to ER stress associated with decreased insulin sensitivity [83,138]. A study investigated the potential role of increased TAG as a trigger for ER stress that could induce insulin insensitivity. XBP-1 knockout mice showed increased expression of ER stress markers but lower TAG levels when compared with wildtype mice after a 1-week fructose diet. Insulin sensitivity in XBP-1 knock-out mice was increased as compared to wildtype mice. From these data it was concluded that ER stress can be disassociated from hepatic insulin resistance supporting the hypothesis that hepatic insulin resistance in models of ER stress may be secondary to ER stress modulation of hepatic lipogenesis [138]. 

Research about fructose induced insulin insensitivity has been strongly focused on the gene expression of metabolic enzymes. The impact of fructose on the hepatic allosterome and thereby on the regulation of enzymes and glucose flux should also be considered for further research.

## 8. Conclusions

After 50 years of research, there is sound evidence that fructose can be qualified as glucogenic and lipogenic sugar. A central role in the hepatic hexose metabolism play the transcription factors ChREBP and SREBP1c, both more potently induced by fructose than by glucose. This seems to reflect the importance of the liver for fructose metabolism. Consequently, fructose consumption may impair glucose homeostasis by increased expression of the gluconeogenic enzymes counteracting the effect of insulin. On the other hand, fructose may lead to an increased expression of lipogenic enzymes with implications on blood TAG and intrahepatic TAG concentrations. 

The impact of fructose on metabolic parameters, as assessed by feeding-trials, is dose-dependent, with probably little effect when low dosages of fructose are used as isocaloric substitution for glucose [139,140,141,142,143]. Similarly, there is a consensus on the TAG raising effects of excessive fructose consumption, but inconsistent data about the effects of a high-fructose energy balanced diet on VLDL secretion and TAG concentrations [139]. While excessive fructose consumption increases IHLC, the effects of high-fructose intake with an energy-balanced diet on liver fat content may need further examination [100,130]. Catalytic amounts of fructose may enhance hepatic glucose uptake via activation of GK, excessive fructose consumption may impair glucose tolerance decreasing GK levels. 

Overall, fructose consumption has been shown to promote hepatic insulin resistance in non-diabetic men [131,133], as well as in rodents [138,144]. Reduced insulin sensitivity with continuous hepatic production and a release of glucose in the fed state is thought to result in long-term hyperglycemia and hyperinsulinemia and to impair muscle insulin sensitivity, and thereby promoting the development of type 2 diabetes mellitus.

However, the debate about the limit between harmless and harmful quantities of fructose is still ongoing, and larger and longer feeding trials are needed in order to complete existing evidence and to increase our understanding of the topic. 

In the future, mathematical models may enhance our understanding of the role of fructose in glucose flux control and will be useful for the design of new experiments [49].
